# Case of Opium Poisoning—Antagonizing Action of Belladonna

**Published:** 1874-03

**Authors:** Ely Van DeWarker

**Affiliations:** Syracuse, N. Y.


### CASE OF OPIUM POISONING—ANTAGONIZING ACTION OF BELLADONNA.

BY ELY VAN DEWARKER, M.D., SYRACUSE, N. Y.

On Saturday, March 30th, I was called to see Eva B., in haste. I reached the house at about 10:30 P. M. I saw the patient, a full sized brunette, aet. sixteen years, lying upon her back in bed, totally unconscious. She was making six or seven respirations per minute in a heavy, labored manner. The limbs were relaxed and cold. The face with a dusky pale appearance. The pupils were greatly contracted. It was impossible to rouse her. The pulse was under fifty, and feeble.

To my mind the case was typical of opium poisoning. I asked if there was laudanum or morphine in the house which the girl might have taken. I was answered there was none. To evacuate the stomach I administered the only emetic I had with me, ipecac and tart. ant. in full doses.

While awaiting the action of the emetic I applied bottles filled with hot water to the feet, and friction to the extremities.

Meanwhile I elicited the following history: The girl had for the previous three days, been troubled with a pain in the side and sore throat and head ache. For the previous four days she had been menstruating in the usual amount and manner. During the evening of Saturday, the sister, Mrs. B., procured medicine from a physician for the pain in Eva's side. The medicine consisted of two powders, one marked and directed to be given first. The first powder, the physician alledges, consisted of Dover's powder and half grain of opium, the second powder to be given in case the first did not have the desired effect, was an ordinary sized Dover's powder. Mrs. B. reached home and gave the first powder as directed, at about nine P. M. The patient says that she soon began to feel dizzy and confused, and undressed and went to bed.

At 9:30 P. M., she was talking with her sister, some minutes later she complained of a strange band-like feeling around the head, what I have before called a "pressure pain," and a sense of drowsiness. Asked for a wet cloth to be placed on her head. Mrs. B. went down stairs and prepared a wet compress, and on returning found her sister insensible. Failing to arouse her, she became alarmed, and I was sent for. This was about ten P. M.

The emetic failing to operate, and seeing the patient no better, I sent for Dr. Terry, who lived near, and he kindly came to my assistance, provided with an electro-magnetic machine, and belladonna and a hypodermic syringe.

On reaching the house, I gave x gtt: Tilden's Fl. Ext. Bel., she swallowed with difficulty. The hot applications and frictions to the extremities continued. In the course of twenty minutes xx gtt. of Bel. again administered.

We now began passing the electro-magnetic current by connecting her hands with the poles of the machine. Under the influence of the current she would move her arms and turn slightly on the bed, but uttered no sound. The current was passed some fifteen minutes. The respirations in this manner could be forced up to eight or ten per minute. On omitting the current, the respirations would drop to six or seven per minute. Dr. Terry, I believe, counted as low as five.

The current was continued with intervals of ten or fifteen minutes until an hour or more had elapsed. The pupils were still contracted, or if dilated, not to that extent to render us positive.

Dr. Terry now suggested the use of the hypodermic syringe, $\frac{1}{30}$ gr. of atropia was accordingly injected into the arm. The pulse at this time was about fifty-five per minute.

In the course of twenty minutes the extremities began to grow warm and the face to flush. In half an hour, more or less, the pulse was about one hundred and fifteen, but the pupil showed but little inclination to dilate.

In an hour or so from the hypodermic injection, the respirations were twelve in a minute, and the patient was more susceptable to the electro-magnetic current, more inclined to resist it. Still unconscious.

The pupils at this stage of the case, according to my vision, were barely responsive.

No more active measures were taken. We passively waited the antagonizing action of the atropia and belladonna.

At three A. M., Dr. Terry returned home. The pupils now responded, but not largely in extent to the antidote. The respirations twelve, the pulse one hundred and twenty, would move the arms voluntarily, still unconscious. Shortly after three A. M., I returned home. About four A. M., she vomited and roused up slightly. At ten A. M., on Sunday, I met Dr. Terry at the bedside. The pulse was one hundred and ten, and the respirations would average fourteen per minute, had roused up and spoken once or twice, and drank water. The pupils were well dilated. She remained in slumber, gradually becoming less profound until the afternoon when she was able to converse. She described her head as feeling bad and dizzy.

On Monday, April first, at ten A. M., the patient was dressed and lying upon the bed. Pulse about eighty. Pupils normal. Respiration normal. Head at times felt dizzy and confused; slight nausea.

On Tuesday, P. M., bowels had not moved, but was comfortable and made no complaint.

As this attack is claimed to be hysterical, I give the following history of the case. At six years old she had a severe attack of scarlitina. Since that sickness she has suffered from a discharge, oftentimes offensive, from her ears and nose. She is large for her age, in good flesh and well developed. Last fall at the begining of one of her menstrual periods she caught cold and suffered a suppression of the discharge. During a period of three days she suffered from great pain in the head, and laid in a partly unconscious state; but at no time was she unable to awswer questions or take food. She told me she had a clear recollection of herself all through that attack, and could answer all questions intelligently. This is the only instance of any trouble during menstruation, which function was established at twelve years of age.

In narrating this case, I have been moved by motives of fairness to all parties interested. It is my single wish to arrive at truth. In carefully arriving at the opinion that it was a clear case of narcotism, I am more inclined to ascribe the untoward result to the peculiarities of the patient than to any excess of the opiate administered.

Aside from the clinical features of the case, which were clearly those of opium poisoning, the behavior of the atropia and extract of belladonna, which were administered by the mouth and hypodermically, was that which usually characterizes the use of this drug as an antidote to opium. Had there not existed some restraining force to the action of the belladonna, we ought to have had a marked degree of atropism developed. On the contrary, the mydriatic effect of the drug was in abayance, so that the pupils were not dilated, but dilatable. Its action upon the vaso-motor nervous system was eminently that of an antagonist of opium poisoning.

In the present state of the mind of the profusion, vibrating between belief and unbelief in the antagonism of belladonna and opium, I deem the case worthy of permanent record.

